# Linking coronary artery disease to neurodegenerative diseases through systems genetics

**DOI:** 10.3389/fgene.2024.1344081

**Published:** 2024-07-25

**Authors:** Martina Vescio, Linda Pattini

**Affiliations:** ^1^ Cardio-Tech Lab, Centro Cardiologico Monzino IRCCS, Milan, Italy; ^2^ Department of Electronics, Information and Bioengineering, Politecnico di Milano, Milan, Italy

**Keywords:** coronary artery disease, systems biology, genetic variants, GWAS, Alzheimer’s disease, Huntington’s disease, network analysis

## Abstract

Coronary artery disease (CAD) is still a leading cause of death worldwide despite the extensive research and the considerable progresses made through the years. As other cardiovascular diseases, CAD is the result of the complex interaction between genetic variants and environmental factors. Currently identified genetic loci associated to CAD revealed the contribution of multiple molecular pathways to its pathogenesis, suggesting the need for a systemic approach to understand the role of genetic determinants. In this study we wanted to investigate how GWAS variants associated to CAD interact with each other and with nearby genes in the context of the coronary artery molecular interactome. GWAS variants associated to CAD were selected from GWAS Catalog, then, a tissue-specific interactome was constructed integrating protein-protein interactions (PPI) from multiple public repositories and computationally inferred co-expression relationships. To focus on the part of the network most relevant for CAD, we selected the interactions connecting the genes carrying a variant associated to the disease. A functional enrichment analysis conducted on the subnetwork revealed that genes carrying genetic variants associated to CAD closely interact with genes related to relevant biological processes, such as extracellular matrix organization, lipoprotein clearance, arterial morphology and inflammatory response. These results confirm that the identified subnetwork reflects the molecular pathways altered in CAD and intercepted by the selected variants. Interestingly, the most connected nodes of the network included amyloid beta precursor protein (APP) and huntingtin (HTT), both implicated in neurodegenerative disorders. In recent years the interest in investigating the common processes between cardiovascular diseases and neurodegenerative disorders is increasing, with growing evidence of a link between CAD and Alzheimer’s disease. The results obtained in this work support the association between such apparently unrelated diseases and highlight the necessity of a systems biology approach to better elucidate shared pathological mechanisms.

## Introduction

Coronary artery disease (CAD) is one of the prominent global contributors to mortality and has seen a steadily increasing in disability-adjusted life year (DALY) since 1990 (Roth et al., 2020), accounting for 8.9 million deaths and 164.0 million DALYs in 2015 (Ralapanawa and Sivakanesan, 2021). The prevalence of CAD varies among different countries, however, CAD related mortality is not a distinctive issue of high-income areas, since it is a major cause of death in countries from all income groups, with a steady increase in developing countries (Ralapanawa and Sivakanesan, 2021). In Western Europe, there were an estimated 9.1 million prevalent cases of CAD in 2015 (Roth et al., 2017).

This condition is characterized by the development of atherosclerosis within the coronary arteries, resulting in an impaired supply of nutrients and oxygen to the myocardium. Atherosclerosis is primarily an inflammatory disorder driven by the interaction between the arterial endothelium and a variety of risk factors, such as dyslipidemia, hypertension and proinflammatory cytokines derived by adipose tissue excess. The inflammation results in cell proliferation, lipids accumulation and extracellular matrix (ECM) production, thickening the arterial walls and forming the characteristic atherosclerotic plaque. These plaques progressively reduce blood flow to the heart muscle, leading to severe consequences, including angina, myocardial infarction (MI), arrhythmias, heart failure, and sudden cardiac death (Libby and Theroux, 2005; Erdmann et al., 2018).

As most cardiovascular diseases, CAD is the result of the complex interplay between environmental, behavioral and genetic factors. It is known that smoking, hyperlipidemia, hypertension, abdominal obesity, and diabetes are related to CAD and to an increased risk of MI (Yusuf et al., 2004; Barquera et al., 2015). Parental history of CAD was also proved to be an independent risk factor, demonstrating the existence of a genetic contribution to the disease (Myers et al., 1990; Marenberg et al., 1994).

Since their development, genome-wide association studies (GWAS) have been employed to investigate the genetic variants related to CAD. These studies were able to identify a large number of common variants with a significant association to the disease (Khera and Kathiresan, 2017; Erdmann et al., 2018).

The evaluation of the impact of such a complex landscape of associated loci and the identification of related pathological mechanisms could benefit from a network approach. In this study we want to investigate the role of variants associated to CAD in the context of a tissue-specific network, to explore how the corresponding genes interact with each other and with their neighbors.

## Materials and methods

### CAD associated variants

Since the aim of the work is to explore CAD genetic variants with a systems biology approach, we retrieved the data available in GWAS Catalog, a public repository of manually curated SNP-trait associations derived from the literature (Sollis et al., 2023). To identify the variants associated with the disease of interest, we selected from the complete GWAS Catalog database only the SNPs associated to one of the following traits: “*Coronary artery disease*”, “*Coronary heart disease*” and “*Ischemic heart disease*”.

To select genetic variants impacting on the final protein product, we retained only missense and nonsense mutations. Without applying additional filters, this research returned results relative to a total of ten different studies carried out from 2011 to 2020, all of which include at least a thousand cases, except for Takeuchi et al. All variants reported have *p*-value <10^−5^.

### Coronary artery interactome construction

**FIGURE 1:**
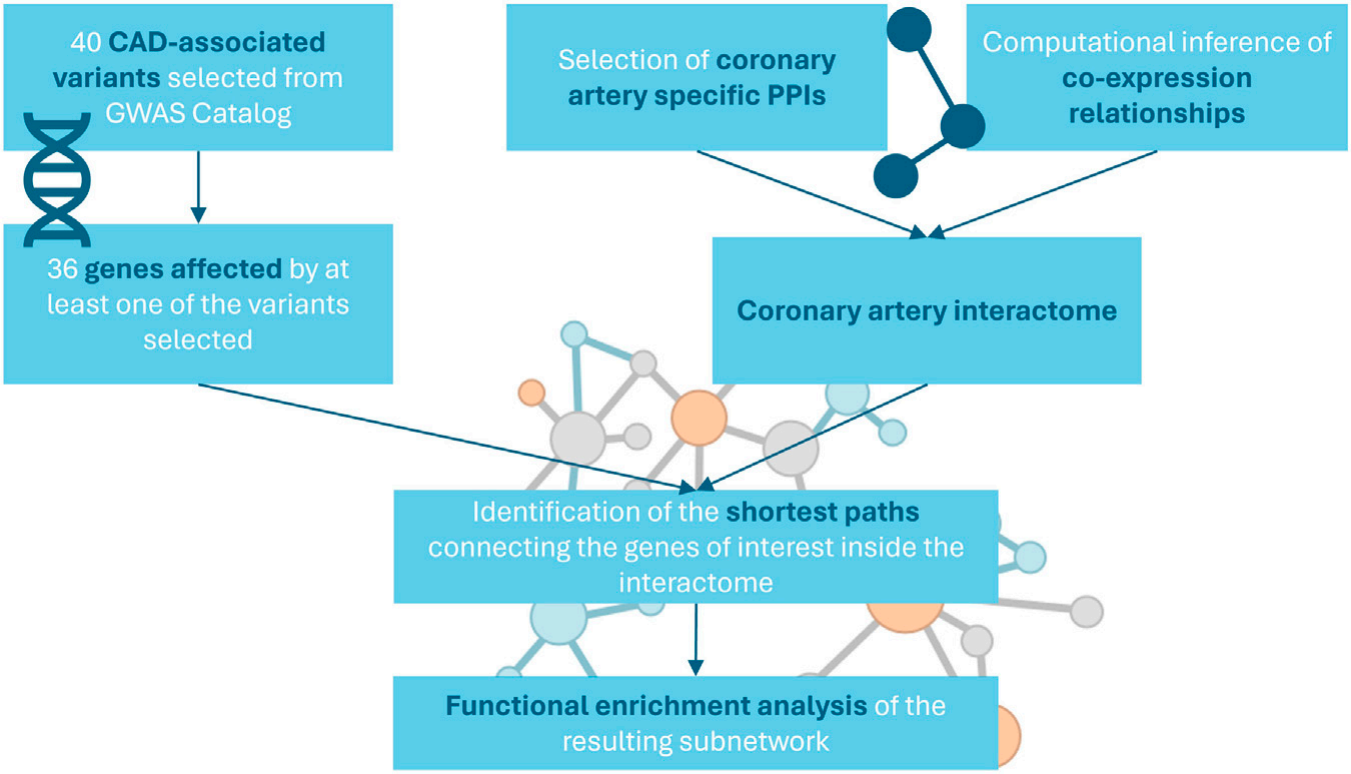
Workflow of the study.

The network specific for coronary artery tissue was constructed by integrating protein-protein interactions (PPIs) from multiple public repositories and computationally inferred co-expression relationships (Figure 1).

First, PPIs were gathered from three recently updated public repositories: STRING (Szklarczyk et al., 2019), that includes physical and functional associations from both high-throughput experiments and computational predictions; mentha (Calderone, Castagnoli and Cesareni, 2013), a manually curated collection of interactions from published literature; and IntAct (Orchard et al., 2014), that contains manually curated experimentally-derived PPIs. We collected all human-specific associations included in the selected databases and filtered them to keep only high confidence interactions, identified by the specific score provided by the single databases (STRING: >0.7, mentha: >0.5, IntAct: >0.45). Scores are computed using different approaches by different databases, however, each one represents the amount of evidence that supports the association in terms of quantity and quality of studies and experiments sourced by the database.

Additionally, we integrated PPIs with the multiple types of human specific associations available in the BioGRID database, a curated database that includes both genetic and protein interactions (Oughtred et al., 2021). We used gene expression data retrieved from the Genotype-Tissue Expression (GTEx) database (Carithers and Moore, 2013), a public repository collecting gene expression data from multiple non-diseased human tissues, to identify genes that appear to be expressed in coronary artery. Gene expression data included RNA-seq data for 240 samples of coronary artery tissue obtained from healthy human donors. Genes were considered as expressed if they had TPM > 0 in at least 80% of the samples included in the dataset. To ensure tissue specificity of the interaction network, we removed associations that included genes not selected as expressed in coronary artery.

Then, GTEx gene expression data were used to estimate the statistical dependencies between expression profiles of genes that included CAD-associated variants and the other genes present in the gene expression dataset. Correlations were computed using a measure of mutual information implemented in the Algorithm for the Reconstruction of Accurate Cellular Networks (ARACNe) (Margolin et al., 2006), an algorithm designed to computationally infer mammalian transcriptional networks from transcriptomics data. ARACNe estimates relationships between expression profiles of different genes as pair-wise mutual information, a measure that allows to capture also non-linear connections. Mutual information is described by the equation:

$$I\left(x,y\right)=S\left(x\right)+S\left(y\right)-S\left(x,y\right)$$

where $S\left(x\right)$ and $S\left(y\right)$ are the entropy of a variable x and y , respectively, and $S\left(x,y\right)$ is the joint entropy of the variables x and y , here representing the gene expression profiles. The statistical associations between genes were considered significant if *p* < 10^−15^ (Percio et al., 2014) since significant thresholds for network construction are conventionally stricter than the standard *p* < 0.05 to account for the high number of connections (Margolin et al., 2006).

### Network analysis

Since we were interested in how genes carrying a genetic variant associated to CAD interact with each other in the context of coronary artery interactome, we focused on the subnetwork constituted by the shortest paths connecting them with each other, as it highlights the core part of the graph responsible for the communication between the genes of interest. The shortest paths were defined as all the alternative paths connecting two selected nodes by intercepting the minimal number of vertices.

To select only the interactions included in the shortest paths connecting each pair of nodes constituted by the genes selected from GWAS Catalog, we applied an unweighted breadth-first search, implemented in the function *all_shortest_paths* included in the R package igraph (Csardi and Nepusz, 2006). The algorithm starts from a source node, explores its immediate neighbor nodes and progressively travels the network by moving to the next level neighbors. The algorithm stops when it reaches the target node.

A functional analysis was carried out on the genes included in the selected subnetwork to identify the biological processes associated to its nodes. The enrichment of gene sets from the hallmark (H), curated (C2) and ontology (C5) collections retrieved from MSigDB (Liberzon et al., 2011) was tested using an over-representation approach, that evaluates the overlap between each gene set and the set of genes of interest (Khatri et al., 2012). The resulting *p*-values were corrected for multiple testing using Benjamini and Hochberg procedure, and the significance threshold was set at FDR < 0.05.

## Results

### The identified subnetwork reflects key processes in CAD

We constructed a tissue-specific heterogeneous network including protein-protein interactions, genetic interactions, and co-expression relationships to model the interactome of the coronary artery. The resulting network consisted of more than 800,000 associations, connecting 18,378 nodes.

To understand their role in the tissue-specific network, we selected the genetic variants reported to be associated to CAD from GWAS Catalog (Supplementary Table S1). The complete list of studies selected from GWAS Catalog is reported in Table 1. All the studies have a solid experimental design and an adequate number of cases and controls, including at least a thousand cases, except for a single study. The selected articles report variants from multiple ethnic groups, allowing to capture a more complete and heterogeneous view of the genetic landscape at the basis of CAD. The final list of variants reported by the selected studies included 40 unique genetic variants mapped to 36 distinct genes. All selected variants have SNP-trait *p*-value <10^−5^. The threshold used is looser than the conventional threshold for genetic studies since the strength of the network approach is the possibility to identify the pathways shared by the different variants, including those who would have a limited individual significance (Wang et al., 2010).

**TABLE 1:** List of articles reporting CAD-associated variants selected from GWAS Catalog.

First author	Date	Journal	Study	Initial sample size	Genotyping technology
van der Harst P	2017-12-06	Circ Res	Identification of 64 Novel Genetic Loci Provides an Expanded View on the Genetic Architecture of Coronary Artery Disease	up to 122,733 cases, up to 424,528 controls	Genome-wide genotyping array
Yamada Y	2018-09-17	Biomed Rep	Identification of 26 novel loci that confer susceptibility to early-onset coronary artery disease in a Japanese population	1,482 Japanese ancestry cases, 5,774 Japanese ancestry controls	Exome genotyping array [Exome array]
Zhou W	2018-08-13	Nat Genet	Efficiently controlling for case-control imbalance and sample relatedness in large-scale genetic association studies	31,355 European ancestry cases, 377,103 European ancestry controls	Genome-wide genotyping array
Takeuchi F	2011-10-05	Eur J Hum Genet	Genome-wide association study of coronary artery disease in the Japanese	806 Japanese ancestry cases, 1,337 Japanese ancestry controls	Genome-wide genotyping array
Dichgans M	2013-11-21	Stroke	Shared genetic susceptibility to ischemic stroke and coronary artery disease: a genome-wide analysis of common variants	33,398 cases, 75,726 controls	Genome-wide genotyping array
Koyama S	2020-10-05	Nat Genet	Population-specific and trans-ancestry genome-wide analyses identify distinct and shared genetic risk loci for coronary artery disease	25,892 Japanese ancestry cases, 95,342 European ancestry cases, 142,336 Japanese ancestry controls, 385,488 European ancestry controls	Genome-wide genotyping array
Matsunaga H	2020-05-29	Circ Genom Precis Med	Transethnic Meta-analysis of Genome-wide Association Studies Identifies Three New Loci and Characterizes Population-specific Differences for Coronary Artery Disease	15,302 Japanese ancestry cases, 76,014 European and East Asian ancestry cases, 36,140 Japanese ancestry controls, 264,785 European and East Asian ancestry controls	Genome-wide genotyping array
Klarin D	2017-07-17	Nat Genet	Genetic analysis in UK Biobank links insulin resistance and transendothelial migration pathways to coronary artery disease	4,831 European ancestry cases, 115,455 European ancestry controls	Genome-wide genotyping array
Schunkert H	2011-03-06	Nat Genet	Large-scale association analysis identifies 13 new susceptibility loci for coronary artery disease	22,233 European ancestry cases, 64,762 European ancestry controls	Genome-wide genotyping array
Nikpay M	2015-09-07	Nat Genet	A comprehensive 1,000 Genomes-based genome-wide association meta-analysis of coronary artery disease	42,096 European ancestry cases, 361 African American cases, 758 Hispanic American cases, 12,658 South Asian ancestry cases, 1,802 Lebanese ancestry cases, 3,614 East Asian ancestry cases, 99,121 European ancestry controls, 2,778 African American controls, 3,337 Hispanic American controls, 12,899 South Asian ancestry controls, 466 Lebanese ancestry controls, 7,709 East Asian ancestry controls	Genome-wide genotyping array

**FIGURE 2:**
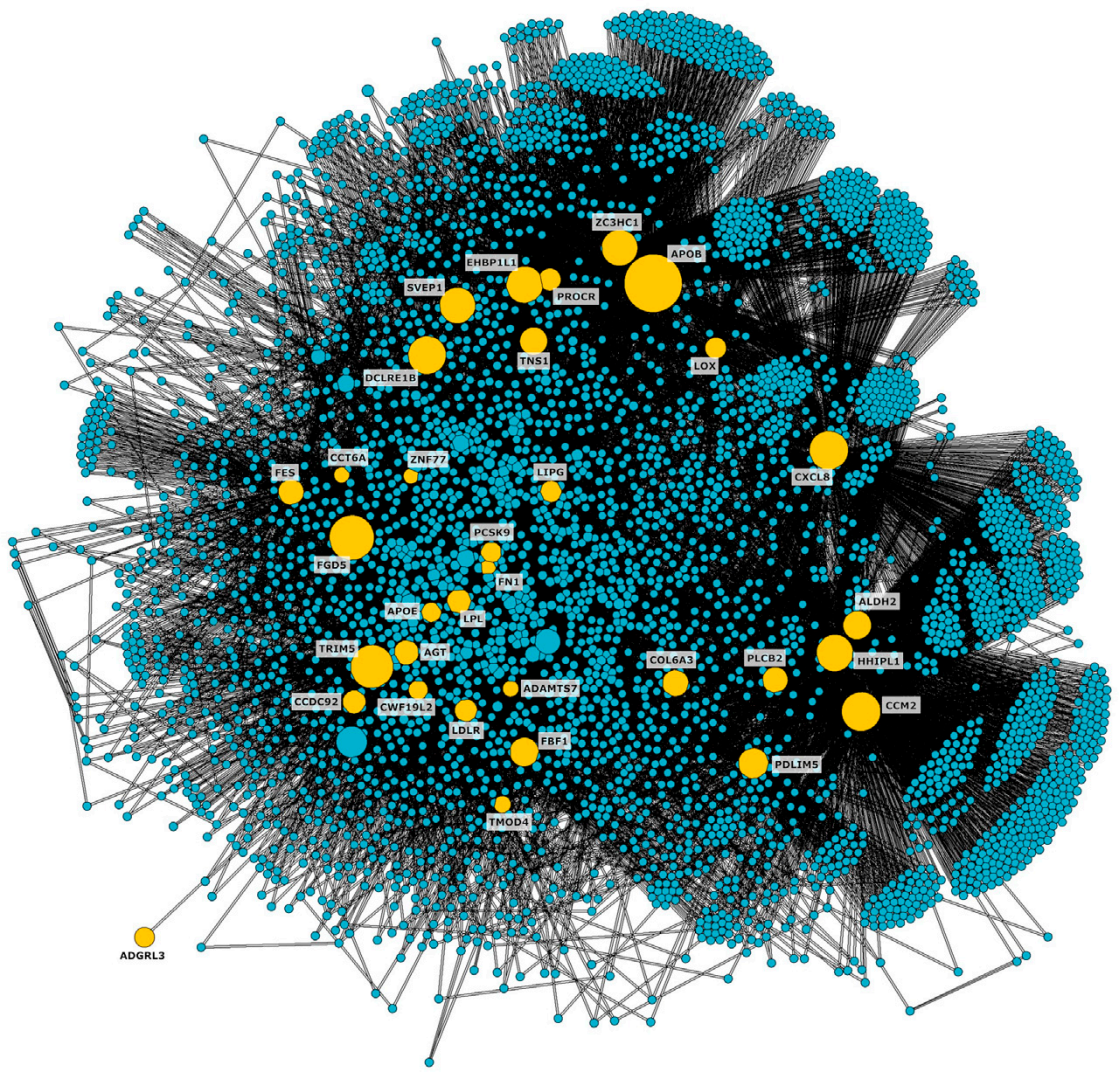
CAD connected subnetwork including the shortest paths connecting nodes affected by genetic variants associated to CAD (shown in yellow).

We located the 36 genes affected by at least one of the variants on the network and identified the shortest paths connecting them with each other. To focus on the portion of the network more relevant to CAD, we isolated the connected subnetwork constituted by the shortest paths, obtaining 4,383 nodes and 49,075 edges (Figure 2).

**FIGURE 3:**
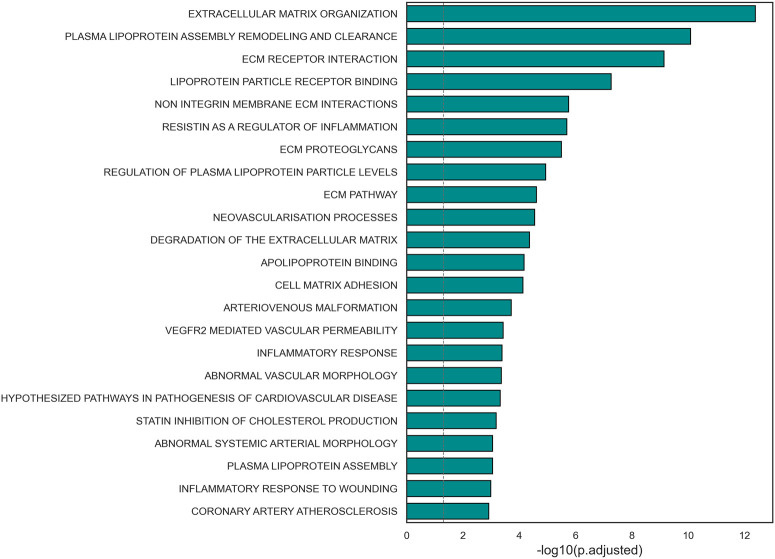
Barplot representation of the statistical significance of terms relevant to CAD pathological mechanisms found significant in the selected subnetwork.

A functional enrichment analysis carried out on the subnetwork confirmed its association with biological processes known to be involved in the pathogenesis of the disease of interest (Figure 3, Supplementary Table S2). Significant terms included extracellular matrix organization, lipoprotein binding and regulation, inflammatory response, and arterial morphology. Additionally, 24 nodes of the subnetwork were included in the gene set *HP_CORONARY_ARTERY_ATHEROSCLEROSIS* (FDR = 0.0012) from Human Phenotype Ontology of MSigDB. Most of these nodes (19 out of 24) were not included in the initial list of variants.

### CAD genes are closely connected to genes related to neurodegenerative diseases

**FIGURE 4:**
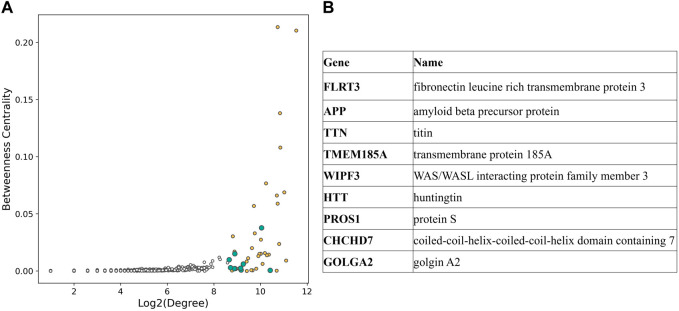
**(A)** Scatterplot displaying degree and betweenness centrality for each node of the CAD subnetwork. Hubs in the top 1% connected nodes are shown with color filled circles, yellow if they are part of the CAD GWAS variants, blue otherwise. **(B)** List of the hubs not included in CAD GWAS variants [blue in **(A)**].

To investigate the pivotal nodes of the subnetwork, we focused on the 1% most connected hubs. The resulting list included 43 genes, and nine of them were not part of the GWAS variants genes (Figure 4).

**FIGURE 5:**
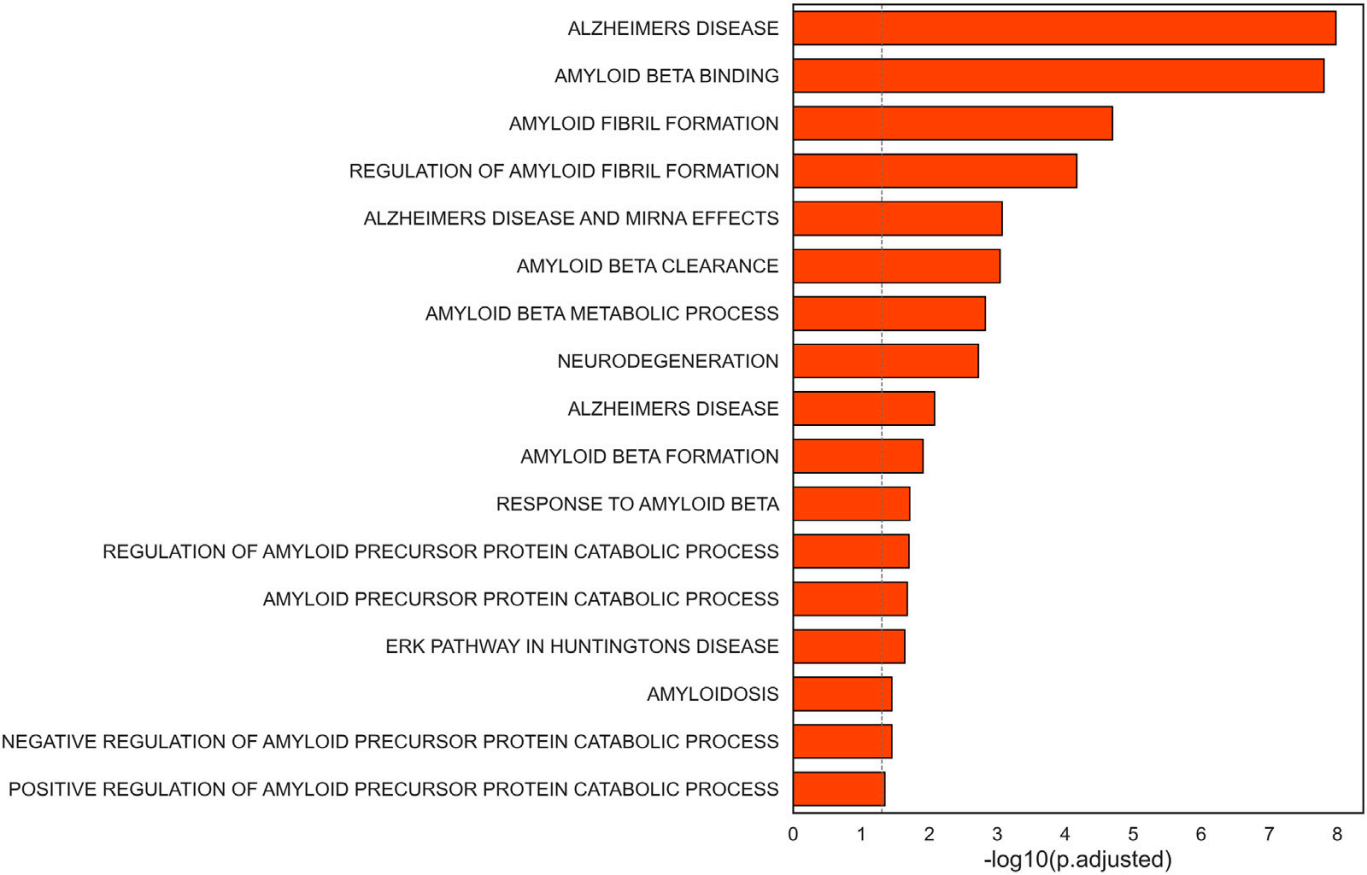
Barplot representation of the statistical significance of terms related to neurodegenerative disorders found significant in the selected subnetwork.

Interestingly, highly connected nodes included APP (amyloid beta precursor protein) and HTT (huntingtin), commonly associated to Alzheimer’s disease and Huntington’s disease, respectively. Additionally, we found multiple gene sets related to neurodegenerative disorders to be enriched in the selected subnetwork for CAD (Figure 5, Supplementary Table S3). This list included terms associated to Alzheimer’s disease, amyloid fibrils formation and binding, neurodegeneration, and pathways in Huntington’s disease.

## Discussion

In this study, we employed a systems biology approach to investigate the role of genetic variants associated to CAD in the context of the coronary artery interactome and to characterize the genes that are closely connected to them.

First, we constructed a tissue-specific network integrating different types of interactions derived from public repositories and computationally inferred. High confidence PPIs were gathered from STRING, mentha and IntAct, additionally, genetic and protein interaction data were collected from BioGRID database. To ensure tissue specificity, gene expression data from coronary artery tissue were obtained from GTEx database and employed to select only interactions that involved genes expressed in the tissue. Then, GTEx data were used to estimate statistical dependencies between variant genes and each other expressed gene in the dataset.

Then, we identified the portion of the coronary artery interactome more relevant for CAD by intercepting the nodes responsible for the communication between variants. Specifically, we selected the subnetwork constituted by the shortest paths connecting the genes that included at least one of the genetic variants associated to CAD in GWAS Catalog.

An enrichment analysis conducted on the subnetwork revealed its association with molecular mechanisms with a critical role in atherosclerosis. For instance, the genes included in the core subnetwork were involved in inflammatory response, a process known to contribute to the formation of the atherosclerotic plaque (Libby and Theroux, 2005; Erdmann et al., 2018). Additionally, the subnetwork shows an overrepresentation of genes associated with lipoprotein metabolism, such as lipoprotein assembly and clearance, lipoprotein levels regulation and regulation of cholesterol production. This further confirms the association between the genes in the subnetwork and key pathological mechanisms in CAD, since the accumulation of lipids in the vascular lumen is widely known as the trigger of the coronary plaque creation (Libby and Theroux, 2005; Erdmann et al., 2018). Another important process in the onset of atherosclerosis is proliferation and remodeling of ECM, leading to the thickening of arterial walls. Terms related to these mechanisms were also found significantly enriched in the subnetwork (Libby and Theroux, 2005; Erdmann et al., 2018).

These results, together with the overrepresentation inside the subnetwork of a term specific for coronary artery atherosclerosis, confirm that the systems biology approach proposed in this work is able to intercept genes relevant to the disease of interest that could have a role in the pathogenesis even in the absence of an established association with CAD.

In the second part of the study, we focused on the pivotal nodes of the subnetwork by selecting the most connected hubs. The list of the 1% most connected nodes included nine genes that were not part of CAD associated genes selected from GWAS Catalog, such as fibronectin leucine rich transmembrane protein 3 (*FLRT3*) and transmembrane protein 185A (*TMEM185A*), broadly expressed transmembrane proteins; titin (*TTN*), known for its involvement in dilated cardiomyopathy (Ware and Cook, 2018); WAS/WASL interacting protein family member 3 (*WIPF3*), a protein that seems to be involved in cytoskeletal dynamics (De Luca et al., 2023); protein S (*PROS1*), a protein responsible for the downregulation of thrombin generation and whose deficiency is associated with thromboembolism (Ten Kate and Van Der Meer, 2008); coiled-coil-helix-coiled-coil-helix domain containing 7 (*CHCHD7*) and golgin A2 (*GOLGA2*).

Interestingly, the most connected hubs also included amyloid beta precursor protein (*APP*), a single pass transmembrane protein expressed in the brain whose accumulation generates the characteristics plaques found in the brains of patients with Alzheimer’s disease (O’Brien and Wong, 2011); and huntingtin (*HTT*), a disease gene linked to Huntington’s disease, a monogenic neurological disorder characterized by the dysfunction and death of neurons (Bates et al., 2015).

The central role of genes linked to neurodegenerative diseases suggests a connection between the CAD-related subnetwork and neurological disorders, that was highlighted by the overrepresentation of genes linked to Alzheimer’s disease, Huntington’s disease, and neurodegeneration.

The link between neurodegenerative disorders and cardiovascular diseases has been under investigation in recent years, with an increasing amount of evidence of an association between heart failure, atrial fibrillation and CAD with an increased incidence of dementia and cognitive impairment (Tini et al., 2020; Zhao et al., 2020; Rivard et al., 2022). The nature of the relationship is still unclear; however, it seems to be maintained even after adjusting for confounding factors, suggesting that the correlation is not simply due to shared risk factors (Rivard et al., 2022).

The relationship between CAD and Alzheimer’s disease is particularly relevant, since patients with severe atherosclerosis show a risk of developing Alzheimer’s disease or vascular dementia that’s three times higher with respect to the general population (Hofman et al., 1997). However, a previous study evaluating their shared genetic architecture failed to find evidence of a causal relevance of CAD for risk of Alzheimer’s disease with the exception of the *APOE* locus (Grace et al., 2018). One possible explanation of this association is the reduction or impairment of cerebral perfusion caused by vascular damage, arterial stenosis and increased arterial stiffness (De La Torre, 2004; Tini et al., 2020).

Furthermore, recent studies suggest that the deposition of amyloid beta (Aβ) peptides, proteolytic fragments of APP, could be the overlapping molecular mechanism between Alzheimer’s disease and CAD. Accumulation of Aβ peptides, besides being an established characteristic of the brain of Alzheimer’s disease patients, was also reported in atherosclerotic plaques. Plasma levels of Aβ1-40 were associated with changes in aortic stiffness, higher carotid intima-media thickness and the severity and extent of arterial damage. Moreover, circulating levels of Aβ1-40 were correlated with the presence of angiographically documented CAD in multiple cohorts (Stakos et al., 2020).

The subnetwork identified in this study supports this hypothesis, since *APP* has a central role as a highly connected hub and the nodes in the subnetwork are strongly enriched of genes involved in the regulation of amyloid beta fibrils formation, Aβ clearance, and catabolic processes of APP.

In conclusion, this work suggests that the application of a systems biology approach can be a valuable tool for the exploration of shared molecular processes between CAD and Alzheimer’s disease, beyond the analysis of single genetic variants (Grace et al., 2018). The employment of a network approach is able to intercept relevant genes without an explicit genetic association with the disease that could be helpful in elucidating interaction mechanisms and discovering new associations with CAD.

## Data Availability

Publicly available datasets were analyzed in this study. This data can be found here: https://www.ebi.ac.uk/gwas/, https://gtexportal.org/home/.

## Abbreviations

GWAS: genome-wide association study; CAD: coronary artery disease; PPI: protein-protein interaction; DALY: disability-adjusted life year; ECM: extracellular matrix; MI: myocardial infarction.

## References

[B1] Barquera S. Pedroza-Tobías A. Medina C. Hernández-Barrera L. Bibbins-Domingo K. Lozano R. (2015). Global overview of the epidemiology of atherosclerotic cardiovascular disease. Archives Med. Res. 46 (5), 328–338. 10.1016/j.arcmed.2015.06.006 26135634

[B2] Bates G. P. Dorsey R. Gusella J. F. Hayden M. R. Kay C. Leavitt B. R. (2015). Huntington disease. Nat. Rev. Dis. Prim. 1 (April), 15005–15021. 10.1038/nrdp.2015.5 27188817

[B3] Calderone A. Castagnoli L. Cesareni G. (2013) ‘Mentha: a resource for browsing integrated protein-interaction networks’, Nat. Methods. Nature Publishing Group, 10(8), pp. 690–691. 10.1038/nmeth.2561 23900247

[B4] Carithers L. J. Moore H. M. (2013). The genotype-tissue expression (GTEx) project. Nat. Genet. 45 (6), 307–308. 10.1089/bio.2015.29031.hmm PMC4010069 23715323

[B5] Csardi G. Nepusz T. (2006). The igraph software package for complex network research. InterJournal, Complex Sy. 1695, 1–9. 10.3724/sp.j.1087.2009.02191

[B6] De La Torre J. C. (2004). Is Alzheimer’s disease a neurodegenerative or a vascular disorder? Data, dogma, and dialectics. Lancet Neurol. 3 (3), 184–190. 10.1016/S1474-4422(04)00683-0 14980533

[B7] De Luca F. Kha M. Swärd K. Johansson M. E. (2023). Identification of ARMH4 and WIPF3 as human podocyte proteins with potential roles in immunomodulation and cytoskeletal dynamics. PLoS ONE 18 (1 January), e0280270. 10.1371/journal.pone.0280270 36649229 PMC9844829

[B8] Erdmann J. Kessler T. Munoz Venegas L. Schunkert H. (2018). A decade of genome-wide association studies for coronary artery disease: the challenges ahead. Cardiovasc. Res. 114 (9), 1241–1257. 10.1093/cvr/cvy084 29617720

[B9] Grace C. Clarke R. Goel A. Farrall M. Watkins H. Hopewell J. C. (2018) ‘Lack of genetic support for shared aetiology of Coronary Artery Disease and Late-onset Alzheimer’s disease’, Sci. Rep. Springer US, 8(1), 7102–7108. 10.1038/s41598-018-25460-2 29740026 PMC5940751

[B10] Hofman A. Ott A. Breteler M. M. Bots M. L. Slooter A. J. van Harskamp F. (1997). Atherosclerosis, apolipoprotein E, and prevalence of dementia and Alzheimer’s disease in the Rotterdam Study. Lancet 349 (9046), 151–154. 10.1016/S0140-6736(96)09328-2 9111537

[B11] Khatri P. Sirota M. Butte A. J. (2012). Ten years of pathway analysis: current approaches and outstanding challenges. PLoS Comput. Biol. 8 (2), e1002375. 10.1371/journal.pcbi.1002375 22383865 PMC3285573

[B12] Khera A. V. Kathiresan S. (2017) ‘Genetics of coronary artery disease: discovery, biology and clinical translation’, Nat. Rev. Genet. Nature Publishing Group, 18(6), 331–344. d 10.1038/nrg.2016.160 28286336 PMC5935119

[B13] Libby P. Theroux P. (2005). Pathophysiology of coronary artery disease. Circulation 111 (25), 3481–3488. 10.1161/CIRCULATIONAHA.105.537878 15983262

[B14] Liberzon A. Subramanian A. Pinchback R. Thorvaldsdóttir H. Tamayo P. Mesirov J. P. (2011). Molecular signatures database (MSigDB) 3.0. Bioinformatics 27, 1739–1740. 10.1093/bioinformatics/btr260 21546393 PMC3106198

[B15] Marenberg M. E. Risch N. Berkman L. F. Floderus B. de Faire U. (1994). Genetic susceptibility to death from coronary heart disease in a study of twins. N. Engl. J. Med. 330, 1041–1046. 10.1056/NEJM199404143301503 8127331

[B16] Margolin A. A. Wang K. Lim W. K. Kustagi M. Nemenman I. Califano A. (2006). Reverse engineering cellular networks. Nat. Protoc. 1 (2), 662–671. 10.1038/nprot.2006.106 17406294

[B17] Myers R. H. Kiely D. K. Cupples L. A. Kannel W. B. (1990). Parental history is an independent risk factor for coronary artery disease: the Framingham Study. Am. Heart J. 120 (4), 963–969. 10.1016/0002-8703(90)90216-K 2220549

[B18] O’Brien R. J. Wong P. C. (2011). Amyloid precursor protein processing and alzheimer’s disease. Annu. Rev. Neurosci. 34, 185–204. 10.1146/annurev-neuro-061010-113613 21456963 PMC3174086

[B19] Orchard S. Ammari M. Aranda B. Breuza L. Briganti L. Broackes-Carter F. (2014). The MIntAct project - IntAct as a common curation platform for 11 molecular interaction databases. Nucleic Acids Res. 42 (D1), 358–363. 10.1093/nar/gkt1115 PMC3965093 24234451

[B20] Oughtred R. Rust J. Chang C. Breitkreutz B. J. Stark C. Willems A. (2021). The BioGRID database: a comprehensive biomedical resource of curated protein, genetic, and chemical interactions. Protein Sci. 30 (1), 187–200. 10.1002/pro.3978 33070389 PMC7737760

[B21] Percio S. Coltella N. Grisanti S. Bernardi R. Pattini L. (2014). A HIF-1 network reveals characteristics of epithelial-mesenchymal transition in acute promyelocytic leukemia. Genome Med. 6 (12), 84. 10.1186/s13073-014-0084-4 25452766 PMC4249615

[B22] Ralapanawa U. Sivakanesan R. (2021). Epidemiology and the magnitude of coronary artery disease and acute coronary syndrome: a narrative review. J. Epidemiol. Glob. Health 11 (2), 169–177. 10.2991/JEGH.K.201217.001 33605111 PMC8242111

[B23] Rivard L. Friberg L. Conen D. Healey J. S. Berge T. Boriani G. (2022). Atrial fibrillation and dementia: a report from the AF-SCREEN international collaboration. Circulation 145 (5), 392–409. 10.1161/CIRCULATIONAHA.121.055018 35100023

[B24] Roth G. A. Johnson C. Abajobir A. Abd-Allah F. Abera S. F. Abyu G. (2017). Global, regional, and national burden of cardiovascular diseases for 10 causes, 1990 to 2015. J. Am. Coll. Cardiol. 70 (1), 1–25. 10.1016/j.jacc.2017.04.052 28527533 PMC5491406

[B25] Roth G. A. Mensah G. A. Johnson C. O. Addolorato G. Ammirati E. Baddour L. M. (2020). Global burden of cardiovascular diseases and risk factors, 1990-2019: update from the GBD 2019 study. J. Am. Coll. Cardiol. 76 (25), 2982–3021. 10.1016/j.jacc.2020.11.010 33309175 PMC7755038

[B26] Sollis E. Mosaku A. Abid A. Buniello A. Cerezo M. Gil L. (2023). The NHGRI-EBI GWAS Catalog: knowledgebase and deposition resource. Nucleic Acids Res. 51 (1 D), D977–D985. 10.1093/nar/gkac1010 36350656 PMC9825413

[B27] Stakos D. A. Stamatelopoulos K. Bampatsias D. Sachse M. Zormpas E. Vlachogiannis N. I. (2020). The alzheimer’s disease amyloid-beta hypothesis in cardiovascular aging and disease: JACC focus seminar. J. Am. Coll. Cardiol. 75 (8), 952–967. 10.1016/j.jacc.2019.12.033 32130931 PMC7042886

[B28] Szklarczyk D. Gable A. L. Lyon D. Junge A. Wyder S. Huerta-Cepas J. (2019) ‘STRING v11: protein-protein association networks with increased coverage, supporting functional discovery in genome-wide experimental datasets’, Nucleic Acids Res. Oxford University Press, 47 (D1), D607–D613. 10.1093/nar/gky1131 30476243 PMC6323986

[B29] Ten Kate M. K. Van Der Meer J. (2008). Protein S deficiency: a clinical perspective. Haemophilia 14 (6), 1222–1228. 10.1111/j.1365-2516.2008.01775.x 18479427

[B30] Tini G. Scagliola R. Monacelli F. La Malfa G. Porto I. Brunelli C. (2020). Alzheimer’s disease and cardiovascular disease: a particular association. Cardiol. Res. Pract. 2020, 2617970. 10.1155/2020/2617970 32454996 PMC7222603

[B31] Wang K. Li M. Hakonarson H. (2010). Analysing biological pathways in genome-wide association studies. Nat. Rev. Genet. Nat. Publ. Group 11 (12), 843–854. 10.1038/nrg2884 21085203

[B32] Ware J. S. Cook S. A. (2018). Role of titin in cardiomyopathy: from DNA variants to patient stratification. Nat. Rev. Cardiol. Nat. Publ. Group 15 (4), 241–252. 10.1038/nrcardio.2017.190 29238064

[B33] Yusuf S. Hawken S. Ounpuu S. Dans T. Avezum A. Lanas F. (2004). Effect of potentially modifiable risk factors associated with myocardial infarction in 52 countries (the INTERHEART study): case-control study. Lancet 364, 937–952. 10.1016/S0140-6736(04)17018-9 15364185

[B34] Zhao E. Lowres N. Woolaston A. Naismith S. L. Gallagher R. (2020). Prevalence and patterns of cognitive impairment in acute coronary syndrome patients: a systematic review. Eur. J. Prev. Cardiol. 27 (3), 284–293. 10.1177/2047487319878945 31645116

